# CeO_2_ Nanoparticle-Containing Polymers for Biomedical Applications: A Review

**DOI:** 10.3390/polym13060924

**Published:** 2021-03-17

**Authors:** Alexander B. Shcherbakov, Vladimir V. Reukov, Alexander V. Yakimansky, Elena L. Krasnopeeva, Olga S. Ivanova, Anton L. Popov, Vladimir K. Ivanov

**Affiliations:** 1 Zabolotny Institute of Microbiology and Virology, National Academy of Sciences of Ukraine, 03680 Kyiv, Ukraine; ceroform@gmail.com; 2 Department of Textiles, Merchandising and Interiors, University of Georgia, Athens, GA, 30602, USA; reukov@uga.edu; 3 Institute of Macromolecular Compounds, Russian Academy of Sciences, 199004 St. Petersburg, Russia; yakimansky@yahoo.com (A.V.Y.); opeeva@gmail.com (E.L.K.); 4 Kurnakov Institute of General and Inorganic Chemistry of the Russian Academy of Sciences, 119991 Moscow, Russia; runetta05@mail.ru (O.S.I.); antonpopovleonid@gmail.com (A.L.P.); 5 Institute of Theoretical and Experimental Biophysics, Russian Academy of Sciences, Pushchino, 142290 Moscow, Russia

**Keywords:** biocomposites, hybrid materials, nanomaterials, therapy, COVID-19

## Abstract

The development of advanced composite biomaterials combining the versatility and biodegradability of polymers and the unique characteristics of metal oxide nanoparticles unveils new horizons in emerging biomedical applications, including tissue regeneration, drug delivery and gene therapy, theranostics and medical imaging. Nanocrystalline cerium(IV) oxide, or nanoceria, stands out from a crowd of other metal oxides as being a truly unique material, showing great potential in biomedicine due to its low systemic toxicity and numerous beneficial effects on living systems. The combination of nanoceria with new generations of biomedical polymers, such as PolyHEMA (poly(2-hydroxyethyl methacrylate)-based hydrogels, electrospun nanofibrous polycaprolactone or natural-based chitosan or cellulose, helps to expand the prospective area of applications by facilitating their bioavailability and averting potential negative effects. This review describes recent advances in biomedical polymeric material practices, highlights up-to-the-minute cerium oxide nanoparticle applications, as well as polymer-nanoceria composites, and aims to address the question: how can nanoceria enhance the biomedical potential of modern polymeric materials?

## 1. Introduction

### 1.1. Polymeric Materials for Biomedical Applications: Advantages and Limitations

**Figure 1:**
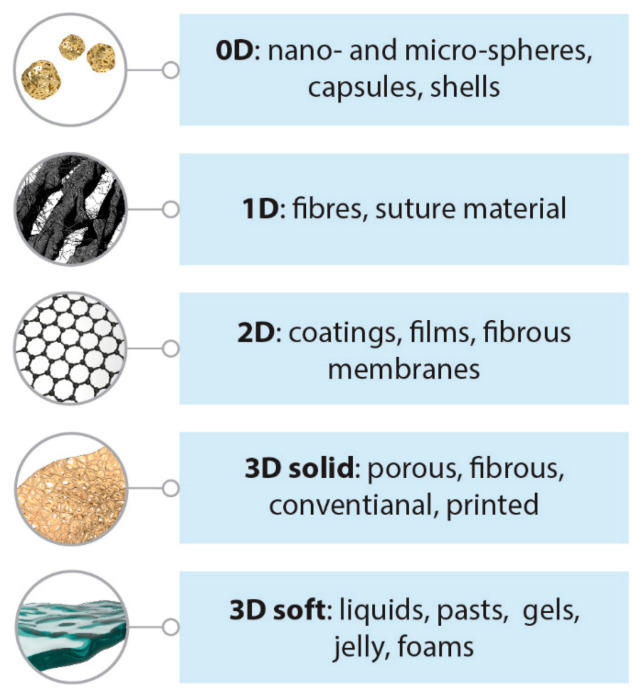
Different dimensionality classes of biomedical polymers.

Polymeric materials have become extremely widespread in various biomedical applications (see Figure 1). The most common synthetic polymers (urethanes, amides, silicones, carbonates, acrylates, vinyl chloride, vinyl alcohol, olefins, esters, etc.) are extensively used in both technical and biomedical fields. Polyurethanes (PU) have numerous applications as materials for vascular grafts, dialysis membranes, artificial heart chambers and valves, dental implants and accessories, artificial heart blood pumps and pacemakers’ wire insulation, etc. Polyamides (PA) are being used for sutures and films; nylon is the material of choice for intravenous and uretic catheters, cannulas and adaptors; semipermeable nylon and hydrophobic polyacrylonitrile (PAN)-based membranes are widely used for kidney dialysis (“artificial kidney”). Polycarbonates (PC) have long been used in dentistry and for medical devices production (blood oxygenators, reservoirs and filter systems, etc.). Acrylic polymers have also been used in dentistry (prosthetic devices, dental implants), as well as in ophthalmology (contact lenses, artificial eyes); acrylic gels are being considered for wound healing, tissue engineering and orthopedic applications. PolyHEMA (poly(2-hydroxyethyl methacrylate) is often considered to be the first hydrogel to be specifically designed as a biomaterial for soft contact lenses. Polycaprolactone (PCL) is useful for long-term implants, bone tissue engineering and controlled drug delivery applications; electrospun fibrous PCL membranes seem to be a good material for wound dressing, etc. In turn, polymeric compositions for cutaneous use and tissue engineering usually contain polymers of a “natural” type-alginate, gelatin, agar-agar, collagen, pectin, chitin and chitosan. Due to their biocompatibility with human tissue, low toxicity, the ability to enhance regenerative processes during wound healing and biodegradability, these natural biopolymers are of particular interest for medicine. Such materials show great promise for creating, for instance, tissue engineering scaffolds; the perspectives of their clinical applications are comprehensively discussed in the literature [1]. Hydrogels based on polysaccharides and their hybrids show great promise for wound healing and cartilage regeneration [2].

Due to its low friction, high wear resistance, toughness, durability, ductility and biocompatibility, an ultra-high molecular weight polyethylene (UHMWPE) has found its way into numerous biomedical applications both in its bulk form, and in the form of coatings [3,4,5], especially as implants (total hip arthroplasty, joint implants, bone tissue engineering). Porous UHMWPE with high osteogenous properties has been successfully used to simulate cancellous-bone tissue [6].

Polymeric materials are being used not only as the components of scaffolds and wound healing patches, but also as promising matrices for bioencapsulation, including hydrogel micro- and nanoparticles, micro- and nano-capsules or films with immobilized biomaterials. The encapsulated materials include both various biologically active substances (proteins including enzymes, DNA, peptides, low molecular weight hormones, antibiotics, drugs etc.) and living cells.

Despite the rapidly expanding use of polymers in medicine, several acute issues remain in this field. For example, when using polymer components for artificial organ development, cases of rejection of these transplants due to foreign body reaction are still common. Problems like bacterial contamination of implantable items (medical devices, artificial limbs and prostheses) also exist, which lead to the formation of bacterial and fungal biofilms. Catheter-related bloodstream infections originate, in some cases, from intravenous catheters, thus shining a light on an important cause of hospital-acquired infections associated with increased mortality and cost. Biofilm formation is not only accompanied by tissue inflammation, but also results in faster erosion of polymeric materials’ surface. Possible sources of the problem can be associated with the use of synthetic biomaterials that tend to have excessive stability in the body (e.g., suture threads or drug carriers) and low biodegradation rate. Some of these problems can be overcome using organic-inorganic composites, multiphase materials where one phase, called the filler, is dispersed in a second continuous phase, called the matrix. Such material combinations combine the advantages exhibited by each component of the material [7,8].

### 1.2. Oxide Fillers for the Modification of the Properties of Polymers

Polymers are good host matrices for various classes of fillers, while filled polymers can acquire significantly improved functional characteristics or new properties in regard to unfilled polymers. Fillers can be divided into three major classes: gaseous (pore forming agents), liquid (rheology modifiers, lubricants, essential oils, drugs, etc.) and solids. The last class of fillers is the most common; the main type of solid fillers is metal oxides.

Owing to their unique properties, metal oxide nanomaterials (TiO_2_, Al_2_O_3_, ZnO, Fe_3_O_4_, Fe_2_O_3_, SiO_2_, ZrO_2_, HfO_2_, etc.) play an important role in a wide range of biomedical applications, such as diagnostics, drug delivery, medical implants, magnetic resonance imaging (MRI), tissue engineering, cancer treatment, etc. [9]. In turn, metal oxide-based polymeric nanocomposites are an extremely important class of modern materials from both a scientific and a technological viewpoint. The potential applications of such nanocomposites are based on their improved chemical, physical (electrical, magnetic, mechanical, optical, rheological, conductive, etc.) and biological properties; they show great promise in certain fields of life sciences, such as biomedicine, agriculture and the environment [10].

Thus, TiO_2_, ZrO_2_ and Al_2_O_3_ have been shown to improve impact strength and fracture toughness, and to decrease the water sorption of dental heat-cured acrylic resins; these metal oxides are also used in preventing denture fractures and undesirable physical changes resulting from oral fluids [11]. Poly(methyl methacrylate) modified with TiO_2_ nanoparticles for stereolithographic complete denture manufacturing seems to be the future in dental care for elderly edentulous patients [12,13]. Nanoparticles of Fe_3_O_4_, Fe_2_O_3_, TiO_2_, ZrO_2_ and Al_2_O_3_ have been used to develop new biomedical polymer hydrogel composites [14] as imaging agents, drug delivery systems, conductive scaffolds and biosensors. Metal oxide-polymer medical coatings play a key role in diversified biomedical devices [15], for example, TiO_2_ enhanced corrosion and wear resistance, as well as bioactivity, to induce hydroxyapatite formation in simulated body fluid [16,17]. Recently, metal oxide nanoparticles-filled polymer materials have been shown to be suitable for biomedical 3D-printing [18].

Common techniques for preparing metal oxide-filled polymer nanocomposites include: (1) polymerization of monomers in the presence of metal oxide nanoparticles in situ; (2) direct blending of metal oxide nanoparticles and polymers by mixing in a melt or solution; (3) the sol-gel process [10]. Originally, polymer composites were reinforced with microsized metal oxide inclusions. Modern scientific level and processing techniques allow the size of fillers to go down to nanoscale, which makes it possible to discover new phenomena and contribute to polymer properties, including those in a biomedical context.

### 1.3. Multifaceted Biomedical Applications of Cerium Oxide Nanoparticles 

Today, cerium oxide nanoparticles (nanoceria, CeNPs) are considered one of the most promising metal oxide nanobiomaterials [19,20,21,22]: pristine (bare) or ligand-stabilized CeNPs are well-known therapeutic agents in regenerative medicine [19,20,21,22] and tissue engineering [22]; they stimulate the proliferation of cells in vitro [21,23,24] and accelerate the healing of lesions in vivo [25], thus reshaping perspectives for wound therapy [26,27]. CeNPs have been shown to be the first inorganic mitogen: in 2019, Popov et al. reported experimental evidence for the mitogenic action of CeNPs in the regeneration of a whole organism, freshwater flatworms [28]. CeNPs can scavenge wound-induced reactive oxygen species, activate gene expression processes and could probably enhance human tissue regeneration, too. Chitosan-coated cerium oxide nanocubes [29], ceria-decorated mesoporous silica nanoparticles [30] and hollow CeNPs with a rough surface [31] have been demonstrated to possess substantial potential for wound healing applications. CeNPs promote wound healing activity in the in vivo animal model [25], as well as the healing of type 2 diabetic foot wounds [32]. Beregova et al. investigated the influence of CeNPs on C-reactive protein and middle-weight molecule concentration in the blood serum of rats in a full-thickness wound model [33]. Due to numerous advantages of CeNPs, such as antioxidant and antimicrobial activities, SOD restoration and other valuable properties Beregova et al. considered CeNPs to be a promising drug for wound healing. Kobyliak and co-workers studied ulcer healing in diabetes and the ability of CeNPs to accelerate the healing process in an animal model [34]. They provided a case report for the successful topical treatment of neuropathic diabetic foot ulcers due to bacteriostatic activity and the anti-inflammatory properties of CeNPs, suggesting the therapeutic potential for topical treatment of such ulcers.

CeNPs can carry out the function of some enzymes (oxidoreductases, phosphatase etc.) and thus belong to the recently discovered family of nanozymes [35]. Due to the rapid development of nanozymes during the last decade, their potential in tissue engineering and regenerative medicine is quite impressive [36,37,38]. Nanozyme-based therapeutic agents are useful for cardiac regeneration, wound healing and neuroprotection [36], they modulate the exercise redox biology of skeletal muscles [39] and efficiently scavenge reactive oxygen species (ROS) in a drug-induced liver injury model [40]. Recently, a nanozyme-reinforced self-protecting hydrogel was shown to promote angiogenesis in an oxidative diabetic wound microenvironment [37].

The experimental observations provided by Kalyanaraman et al. have provided a solid base through studies of biocompatibility and toxicity data on CeNPs, including 28-day systemic toxicity and genotoxicity studies performed according to current regulatory standards [41]. The results have shown that local tissue reactions caused by CeNPs were very low (implantation irritation index of less than 3) and thus CeNPs were tolerated better than most other implant materials tested. Furthermore, CeNPs demonstrated virtually no systemic toxicity or in vivo micronucleus induction in bone marrow *via* the implantation route [41].

Significant challenges remain in the development of tissue-engineered cartilages: scaffold materials often lead to significant inflammatory reactions. The results obtained by Nicole Rotter and colleagues suggested that inflammatory reactions against widespread scaffold materials (polyglycolic acid-polylactic acid copolymer) lead to the production of cytokines such as IL-1α that may inhibit cartilage tissue formation in autologous transplant models [42]. CeNPs possess anti-inflammatory properties [19], and it has been shown in vitro (macrophages, RAW264.7 cells) and in vivo (Sprague-Dawley rats) that treatment with CeNPs reduces both the level of IL-6 and inflammatory markers in mice blood and the activation of p65-nuclear factor-κB (NF-κB) in cultured RAW264.7 cells [43]. CeNPs also reduce levels of cytokines TNF-α, IL-1α and IL-1β [44]. Ponnurangam et al. demonstrated the high potential of CeNPs in improving articular cartilage tissue properties and their long-term inflammatory reaction. Rod-like CeNPs (particle dimensions 65 ± 10 nm and 8 ± 1 nm, concentration range 100–1000 μg/mL) were found to be biocompatible with chondrocytes and improved the mechanical and biochemical properties of engineered cartilage when embedded in the agarose scaffold. Introducing CeNPs into a biological repair strategy thus may serve as a means of improving engineered cartilage properties in vitro prior to implantation [45]. The incorporation of CeO_2_ in hydroxyapatite coatings for orthopedic implants can be a good strategy not only to promote osseointegration, but also to reduce implant-induced inflammatory reactions [46]. Prefac et al. showed the perspective of using CeNPs-containing thin films as bioactive coatings for orthopedic implants [47]. According to recent analysis [19], the ability of cerium compounds (including CeNPs) to decrease the activity of the reticuloendothelial system can be highly promising in prosthesis or tissue engineered constructs implantation, significantly reducing graft rejection rates. 

CeNPs efficiently scavenge ROS and free radicals, which makes them a promising antioxidant protective material. Unfortunately, free CeNPs are easily excreted from the organism or can be dissolved to form toxic species [48]. Moreover, despite their proven antioxidant activity, CeNPs can cause oxidative stress under certain conditions [19]. Thus, there is an urgent need to develop improved strategies to enhance the performance of CeNPs, especially by binding them to a matrix to improve their antioxidant properties and decrease toxicity. Phagocytosis of nanoceria by cells can result in significant cytotoxic effects, thus limiting the applicability of CeNPs as an antioxidant in biomedicine. Recently, Weaver et al. demonstrated that this cytotoxicity can be eliminated by embedding CeNPs into a polymer (e.g., hydrogel) matrix [49]. The rational design of polymer-CeNPs composites would not only allow modulation of the anti/pro-oxidant activity of CeO_2_ nanoparticles, but also enhance the antimicrobial and antioxidative properties of this material [50].

### 1.4. Cerium Oxide Nanoparticles as a Modifier of Polymer Properties 

The incorporation of ceria nanoparticles into polymeric materials enables the enhancement of the mechanical properties and thermal stability of composites. As an example, poly(ether-ether) and poly(ether-ester) block copolymers have widely been applied in biomedicine due to their good safety and biocompatibility [51]; the introduction of CeO_2_ nanoparticles into poly(ether-ester) enhances the mechanical, thermal and low-temperature elastic recovery properties and photostability of these polymers [52]. In turn, due to its high-performance characteristics, thermoplastic polyurethane (TPU) is widely used in advanced medical and healthcare products, such as diagnostic devices, artificial respiration units, healthcare mattresses, dental materials, compression stockings, medical instrument cables, gel shoe orthotics and wound dressings, etc. CeNPs form coordination bonds with TPU, which leads to an increase in TPU’s microphase separation degree and, in addition, significantly improves surface properties and biological compatibility, including blood compatibility and chemical resistance. Meanwhile, TPU’s mechanical and thermal properties are also enhanced dramatically upon CeNPs’ introduction [53]. CeNPs acting as a crosslinking agent for collagen has invoked considerable interest as a potential modifier to increase protein thermal stability [54].

The development of CeO_2_-based polymer composites seems to be quite useful for the enhancement of the properties of both ceria and polymers. A current literature survey has shown that ceria nanoparticles are widely used as polymer fillers, e.g. for the construction of advanced new functional materials for biomedical applications. This paper is a first attempt at summarizing current data on the multifaceted biomedical applications of CeO_2_ nanoparticles-containing polymers. The most notable examples of polymeric CeNPs-containing materials and the biomedical effects achieved are discussed below and shown in Table 1.

## 2. Ceria-Containing Tissue Engineering Scaffolds

Tissue engineering scaffolds are typically three-dimensional biomaterials that replace or repair whole tissues or their parts (e.g., bone, cartilage, blood vessel, bladder, skin, muscle, etc.) in the body. Such tissue engineering materials can be categorized into three main groups: porous scaffolds, fibrous scaffolds and hydrogels. 

### 2.1. Porous Scaffolds

Bones are the second-most transplanted tissue, after blood transfusion, and an increased demand for bone grafts has led to the mass production of synthetic scaffold alternatives [60]. To replace bone tissue, rigid porous materials are often used; for example, porous UHMWPE has been used to simulate cancellous tissue [6]. As a replacement for the organic component of natural bone (collagen), the use of chitosan, chitin and gelatin, as well as various types of celluloses (cotton, microcrystalline, bacterial, etc.), has been proposed [82]. Partial replacement or modification of calcium phosphates with cerium compounds, primarily with cerium oxide nanoparticles, which have a very important biological role in accelerating bone tissue repair, seems to be quite promising [83].

### 2.2. Electrospun Fibers

**Figure 2:**
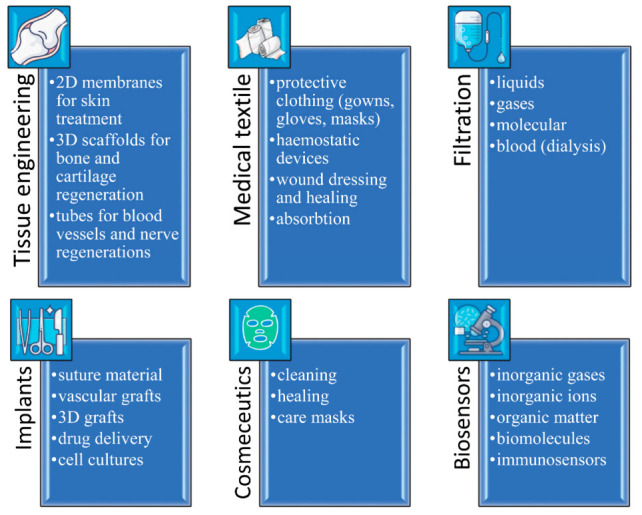
Biomedical applications of polymeric electrospun fibers.

Fibrous polymeric structures are of great importance due to their morphology being similar to that of an extracellular matrix. A number of technologies are used to fabricate fibrous scaffolds, including knitting, weaving, braiding, nonwoven production (self-assembly, phase separation) and spinning, with electrostatic spinning (electrospinning) being the most popular approach to vascular, neural, bone, cartilage and tendon/ligament tissue engineering. The major fields of the biomedical application of polymeric electrospun fibers are shown in Figure 2.

Electrospun fibrous scaffolds contain interconnecting pores, thus resembling a natural extracellular matrix and showing a high potential to facilitate the formation of artificial functional tissues [84]. Augustine et al. developed electrospun polycaprolactone (PCL)-based tissue-engineering scaffolds loaded with CeNPs and evaluated their morphological and physico-mechanical features. In vitro and in vivo studies were performed to show the ability of CeNPs-containing scaffolds to enhance cell adhesion and angiogenesis; the studies confirmed that these scaffolds supported cell adhesion and angiogenesis better than bare polymeric scaffolds [55]. Jain et al. reported the fabrication of CeNPs-decorated PCL and PCL-gelatin blend (PCLG) nanofibers by electrospinning [85]. CeNPs-based PCLG scaffolds were found to be cytocompatible with various cell types, including primary cardiomyocytes, showing a marked decrease in ROS levels when subjected to H_2_O_2_-induced oxidative stress. Interestingly, it was found that CeNPs-PCLG nanofibers suppressed agonist-induced cardiac hypertrophy, demonstrating its promise as a cardiac patch with antioxidant and anti-hypertrophic properties. Using electrospinning, Narruddin et al. fabricated and studied nanocomposites of polycaprolactone (PCL), containing different ratios of gamat oil and cerium oxide particles, that can be used in the healing of wounds. The obtained nanocomposites increased hydrophobicity, improved tensile strength and demonstrated physicochemical properties that are well suited for wound healing applications [86].

### 2.3. Composite 2D Films and 3D Scaffolds

Mandoli et al. induced the growth of aligned stem cells using hybrid 2D poly(D, L-lactic-co-glycolic acid) (PLGA)-CeNPs composite films [78]. CeNPs’ incorporation profoundly affected the scaffolds’ mechanical, topographical and biological properties. This ability helped to produce films to host and support cell growth; these films were evaluated after 1, 3 and 6 days of culturing with murine-derived cardiac and mesenchymal stem cells (CSCs and MSCs). The CeNPs composite films were then compared with bare PLGA films without nanoparticles and with films loaded with nanoparticulate TiO_2_. Aligned cell growth was observed only for the scaffolds that incorporated oriented ceria nanoparticles, attributed to the CeNPs’ ability to modulate the roughness pitch, thus improving cell sensitivity towards the host surface features. Improved CSCs’ and MSCs’ proliferative activities were observed for CeO_2_ composites in comparison with either TiO_2_-modified or unfilled PLGA films. This effect is supposedly related to CeO_2_ antioxidative properties [78].

Dulany et al. developed a functional synthetic 3D scaffold for bone engineering based on (1,8-octanediol-co-citrate), beta-tricalcium phosphate and CeNPs [60]. They studied cellular and tissue compatibility of the scaffold to assess the interaction of nanocomposites with both human osteoblast cells and rat subcutaneous tissue. The scaffolds were shown to be biocompatible in both in vitro and in vivo models and supported the attachment, proliferation, mineralization and infiltration of cells. Using hydrogen peroxide, Dulany et al. simulated oxidative stress to study the protective properties of nanocomposite scaffolds, to demonstrate the reduction of cytotoxicity and restoration of osteoblast cell mineralization in vitro. They also found that, after in vivo implantation, the scaffolds exhibited high biocompatibility required for successful bone engineering. The cells could penetrate the scaffold, the surrounding tissues elicited minimal immune response and, after 30 days of implantation, scaffold degradation was observed [60]. 

A three-dimensional composite scaffold made from cellulose and CeNPs was prepared by lyophilization [48]. Cubical nanoceria particles, in the size range 3.2–32 nm, were successfully immobilized into the cellulose matrix without agglomeration and exhibited excellent antioxidant properties in a pH-dependent manner. Such composite nanomaterial has shown promise in clinical applications as an effective antioxidative green material for scavenging reactive oxygen species.

Cerium valence states drastically affect cell behavior on the scaffold surface [56,87]. Mesenchymal stem (MSCs) and osteoblast-like (MG63) cells were cultured on PL/CNP surfaces enriched with either Ce^4+^ or Ce^3+^ regions. Different surface valence state regions either promoted or inhibited cell spreading, migration and adhesion behavior, resulting in rapid or slow cell proliferation [56].

## 3. Wound Dressings and Other Topical Applications for Ceria-Containing Polymers

Skin is the outer covering of the human body and, as the largest organ, plays an essential role as a barrier to the external environment. Major types of skin wounds include ulcers (pressure, diabetic, venous) and burns. Wound dressings are widely used to prevent interruption of the normal wound healing process and to stimulate it. Conventional wound dressings (cotton wool, gauze or mesh, natural or synthetic bandages, etc.) are gradually being replaced by modern ones (hydrocolloids, alginates, hydrogels, semi-permeable adhesive films, foams, biological dressings and fabric skin substitutes). The main principles of an “ideal” wound dressing and existing types of commercially available synthetic dressings have been comprehensively discussed in a recent review [88].

CeNPs were demonstrated to improve the rate of wound healing and to decrease the severity of the treatment process [19] (see above). Ceria-containing nanocomposites with biopolymers are commonly used in cell technologies to obtain biocompatible matrices providing good adhesion to mesenchymal stem cells; the introduction of CeNPs as an inorganic component significantly increases the rate of cell proliferation and differentiation [20]. Chigurupati et al. showed that the topical application of CeNPs accelerates the healing of full-thickness dermal wounds in mice by enhancement of the proliferation and migration of fibroblasts, keratinocytes and vascular endothelial cells (VECs) [23]. Obviously, the use of CeNPs as a filler for wound dressing materials has great potential. 

### 3.1. Electrospun Fibrous Membranes

Electrospun fibrous membranes seem to be a good material for cutaneous substitutes and wound dressings [89]. Augustine et al. proposed CeNPs-modified electrospun poly(3-hydroxybutyrate-co-3-hydroxyvalerate) membranes for diabetic wound healing applications; with these membranes, cell proliferation and healing rates were shown to increase both in vitro and in vivo [63].

Naseri-Nosar et al. incorporated CeNPs into electrospun poly(ε-caprolactone)/gelatin films [65]. Their study showed that, after 2 weeks’ treatment, a CeO_2_ nanoparticle-containing dressing promoted nearly 100% wound closure compared to a treatment with sterile gauze which achieved only 63% wound closure. Rather et al. studied CeNPs-functionalized PCL-gelatin electrospun fiber mats for wound healing applications [66]. The mesh obtained exhibited superoxide dismutase (SOD) mimetic activity and enhanced proliferation of 3T3-L1 cells by ∼48%, as confirmed by alamarBlue^®^ Assay and SEM images of the cells grown on the nanofibrous material. Hu et al. incorporated CeO_2_ nanorods in tissue engineering electrospun polyvinyl alcohol mats to prevent their biofouling during long-term usage, typically resulting in persistent infections and device damage [90].

### 3.2. 2D Films and Composites

Hao et al. developed and evaluated the functional characteristics of a CeNPs-loaded polycaprolactone (PCL) dressing in cutaneous wound healing in nursing care [69]. The robust CeNPs-PCL dressing showed high antimicrobial activity against *E. coli* and *S. aureus*, a strong wound healing effect and good biocompatibility. The addition of bioflavonoid curcumin in CeNPs-poly(acrylamide) dressing material enhanced the healing effect [50]. 

The most widely used natural film-forming biopolymers are α- (starch, dextran) or β- (cellulose) polyglucans and anionic polysaccharides (pectin, alginate), proteins (gelatin, elastin, collagen) and cationic chitosan derivatives. In order to improve the antibacterial, thermal and mechanical properties of the polymer composite material, Kalaycioğlu et al. loaded a chitosan/cellulose acetate film with nanosized cerium oxide [64]. The physical properties and antibacterial characteristics of the films obtained showed promise as a wound dressing material. 

## 4. Ceria-Containing Gel-Like Polymeric Materials

**Figure 3:**
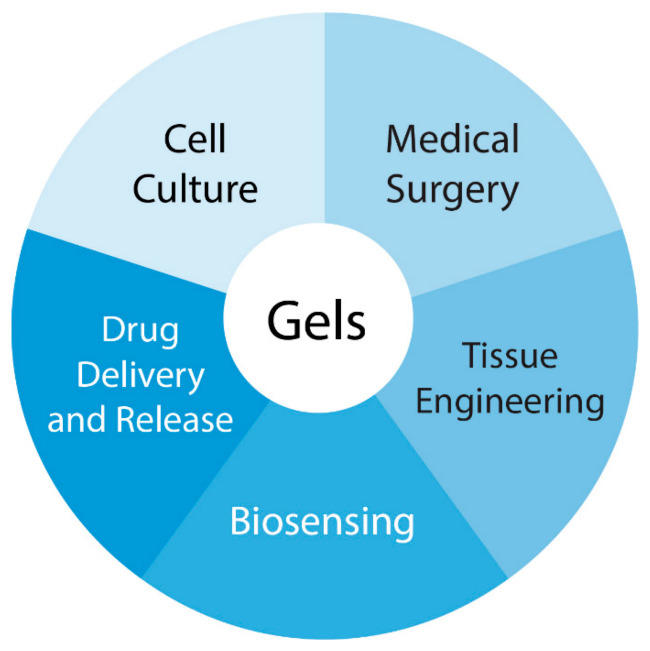
Applications of hydrogels in biomedicine.

Compared with any other polymeric materials being used for biomedical purposes, hydrogels are similar in nature to living tissue and extracellular matrix components, providing a suitable environment for cell survival and growth. Gel-like polymeric materials are widely used in medicine, see Figure 3; this type of material has a number of advantages over conventional sorption wound dressings (gauze, cotton, non-woven materials and polymer sponges), for which a dynamic balance of microflora concentration is established at the “dressing-wound” interface. Gel-like dressings provide a plasticizing effect on wound tissues, soften necrotic formations, facilitate the mechanical removal of non-viable tissues, and prevent the development of infection on the surface of the wound under the scab. They maintain a moist environment in the wound that is optimal for the normal course of regeneration processes. The hydrogel adheres well to the skin around the wound and it can be easily and painlessly replaced when applying dressings. Encapsulating, microporous or injectable hydrogels provide a regenerative medicine platform due to their ability to create an environment that supports transplanted or endogenous infiltrating cells and enable these cells to restore or replace the function of tissues lost due to disease or trauma [91]. 

Raja and Fathima engineered a genipin cross-linked gelatin hydrogel composite containing an optimized concentration of CeNPs for wound healing [67]. In vivo experiments on wound healing and further histological examination demonstrated that the rats treated with this hydrogel showed a greater infiltration of leukocytes and a larger deposition of collagen when compared to pristine gelatin and control groups and were fully recovered in 12 days. 

Using CeNPs, Wu et al. developed a “seed-and-soil” strategy by simultaneously reshaping the oxidative wound microenvironment into a pro-regenerative one (the “soil”) and providing proangiogenic microRNA cues (the “seed”) via a microRNA-impregnated, redox-modulatory, nanozyme-reinforced, self-protecting hydrogel (PCN-miR/Col) [37]. Due to CeNPs, the PCN-miR/Col not only reshapes the hostile oxidative wound microenvironment, but also ensures the structural integrity of the encapsulated proangiogenic miRNA in the oxidative microenvironment. Diabetic wounds treated with the PCN-miR/Col display a remarkably accelerated wound closure and an enhanced quality of the healed wound as demonstrated by the highly ordered alignment of collagen fibers, skin appendage morphogenesis, functional new blood vessel growth and oxygen saturation. Sener and co-workers designed zwitterionic cryogels (gels formed at below-freezing temperatures) loaded with CeNPs-microRNA miR-146a that were topically applicable, injectable and self-healable, and that provided a sustained release of the therapeutic molecules. These cryogels demonstrated high efficacy and enhanced viability in vivo in a diabetic mouse wound healing model [70].

Bhattacharya et al. designed a multicomponent poly(acrylamide) hydrogel dressing containing CeNPs and curcumin (ACC) [50]. In a full-thickness acute wound healing model of a rat, a single application of this type of dressing demonstrated high wound healing efficacy (78%) and negligible scarring in seven days. Enhanced cell proliferation, a higher collagen content, advanced wound maturity, re-epithelialization and granulation tissue formation were observed. The study of cellular mechanisms of wound healing identified MCP-1 and TGF-β as the key drivers of differential and accelerated healing observed in the ACC group. The upregulation of growth-related signaling pathways (HER2/ErbB2, TGF-β-Smad2/3, MAPK/ERK, AKT and VEGF) promoted almost scarless healing in animals treated with an ACC hydrogel dressing. The combination of polymer, curcumin and CeNPs used in the study demonstrated a distinct advantage and proved to be a better agent for complete wound healing.

Weaver and Stabler proposed a CeNPs-containing alginate hydrogel for cellular transplantation; this composite hydrogel provided cytoprotection for encapsulated beta cells from free radical attack without any signs of cytotoxicity, even up to 10 mM concentration [49]. Citrate-stabilized CeNPs integrated into a polysaccharide hydrogel matrix containing natural and synthetic polysaccharide polymers (pectin, alginate, chitosan, agar-agar, water-soluble cellulose derivatives) were used as the therapeutic agent for wound healing in vivo (male Wistar rats) [92]. The study showed that this type of dressing enhanced wound healing by inhibiting the inflammatory response and accelerating the development of fibroblasts, with an early onset of new collagen synthesis, which led to the formation of more tender postoperative scars.

## 5. Polymeric Composites with Ceria Nanoparticles for Drug Delivery and Gene Therapy

**Figure 4:**
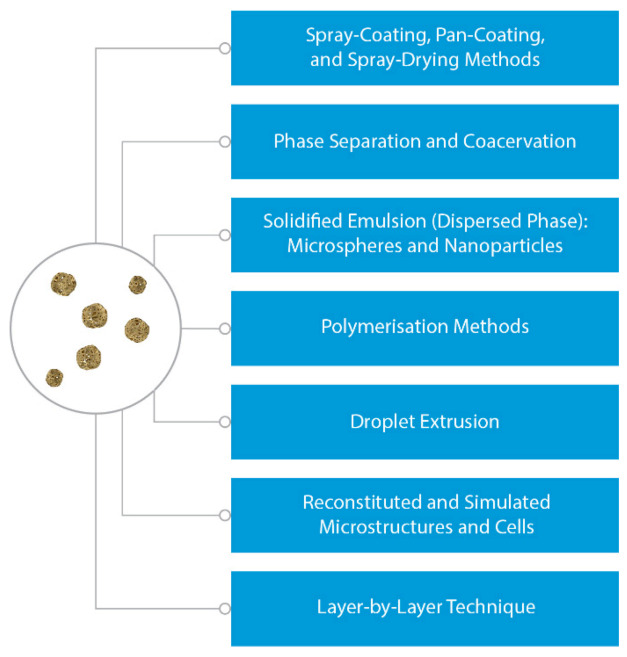
Classification of microencapsulation techniques used in preparation of medicinal products.

Microencapsulation methods used in the preparation of polymeric medicinal products [93], including the LbL technique, are listed in Figure 4.

### 5.1. Polymeric Micro- and Nanoparticles

A large group of ceria-based composite materials is represented by nano- and microparticles of polymeric materials containing nanoparticles of cerium oxide. Such polymeric composite CeNPs-filled materials seem to be useful for advanced drug delivery [56] and gene therapy [94]. Upon selecting a carrier for drug delivery, the ability of metal oxide nanoparticles to induce apoptosis of cells in vitro and an inflammatory response in rats in vivo has been investigated [95]. Unlike other nanoparticles studied, autophagy and apoptosis were not detected 12 h after the exposure of cells to 100 μg/mL of CeNPs. The autophagy related genes LC3, atg5, beclin1 and bcl2 were not changed in protein level at 0 to 200 μg/mL of CeNPs. It was shown in vivo that only four of 27 cytokines (IL12P70, RANTES, IL-X and MIP-1a) were changed on day 28 after exposure to CeNPs. Yang et al. suggested CeNPs to be the ideal vehicle for targeted drug delivery both in vivo and in vitro [95].

Chitosan derivatives, e.g., trimethyl chitosan (TMC), are widely used in drug delivery applications [96]; Mohammad et al. combined the oxidoreductive potential of CeNPs with the antimicrobial, antioxidative and biosorptive properties of TMC [97]. In vitro antibacterial and antioxidative assays proved that CeNPs incorporated into a TMC biopolymer significantly enhanced the efficiency of the latter by increasing interaction with the bacterial cell wall and serving as the free radical scavenger. 

Gene therapy is a good approach to the treatment of genetic and acquired diseases. The introduction of a foreign gene into the cell genotype (transfection) is used in biotechnology (production of valuable recombinant proteins and peptides), medicine (gene therapy) and agriculture. CeNPs-containing polymeric materials can be considered as promising candidates for the design of DNA-containing carriers to produce genetically modified cells, both because of biocompatibility, low toxicity and biodegradability, and because of their ability to penetrate easily through biological interfaces (membranes) and deliver bound pharmaceuticals intracellularly. In this way, chitosan directly forms complexes with negatively charged DNA molecules due to the positive charge of the polymer, while for the other polymers such DNA complexes can be formed indirectly, via binding with inorganic nanoparticles, for example, positively charged nanoparticles of cerium oxide. It is well known that cerium compounds (including CeNPs) can perform the function of a restriction enzyme [19] and actively promote transfection in gene therapy [19,98]. Hasanzadeh et al. synthesized CeNPs-containing polyethylenimine (PEI) nanoparticles (124 ± 7 nm, ζ-potential 22 ± 2 mV) as a novel and promising gene delivery vector pDNA-PEI-CeO_2_ [99]. In both WEHI 164 cancer cells and L929 normal cells, the cytotoxicity of pDNA-PEI-CeO_2_ nanoparticles was less than the cytotoxicity of pDNA-PEI, while the former nanocomposite demonstrated enhanced gene transfection and higher cytotoxicity in cancer cells than in normal cells. Gao et al. studied CeNPs-loaded poly(lactide-co-glycolide)-polyethyleneglycol (PEG/PLGA) particles (~40 nm) and their role in blood-brain barrier permeability in cerebral ischemia [75]. They showed that this nanomaterial was highly promising for cerebral ischemic therapy, due to its neuroprotective properties; CeO_2_ nanoparticles combined with PEG/PLGA matrices exhibited greater efficacy, resulting in a 60% lessening of focal ischemia and a 78% decrease in a brain edema, in comparison with the control animals. RNA delivery vectors have been shown to benefit from an aptamer targeting EGFR and CeNPs + PEI composites have been shown to provide simultaneous antioxidant and gene therapy [94]. 

Since polymeric nano- and microparticles (for example, those made of cationic chitosan and its derivatives) can efficiently be absorbed on mucous membranes, they can be used to create oral and nasal prolonged-release vaccines. For example, vaccines encapsulated in polymer matrices are released over a long time period (1–6 months) and thus re-immunization is not required. At present, intense research is underway to create vaccines encapsulated in chitosan-containing polymer matrices; a variety of antigens is used, ranging from diphtheria and cholera toxin and ending with HIV and SARS-CoV-2. Considering that cerium oxide nanoparticles possess adjuvant properties and can significantly enhance the immune response of vaccines (for example, the anti-influenza vaccine [100]), CeNPs are candidate inorganic components for this type of polymeric medical material.

### 5.2. Layer-by-Layer Polyelectrolyte Coatings and Capsules

Another important group of microcarriers suitable for transporting biologically active substances and cellular elements is multilayer polymer microcapsules. A layer-by-layer (LbL) method of synthesizing polyelectrolyte coatings and microcapsules is based on the use of differently charged polyelectrolytes that are alternately adsorbed on an organic (polymeric) or inorganic (oxides, calcium carbonate) substrate. Polymeric polyelectrolyte microcapsules are one of the most promising means for the effective controlled delivery of substances to target organs and tissues, while LbL shells are very useful for the creation of multipurpose “smart” protective and shielding coatings. In their focused paper, Popov and co-workers predicted great prospects for the use of cerium dioxide nanoparticles in polymer coatings and microcapsules produced by LbL self-assembly [101]. Modification of polyelectrolyte microcapsules with CeNPs appears to be a promising method for the design of synergistic pharmaceuticals based on ceria nanomaterials and anticancer drugs with localization and release control. CeNPs can be introduced into the microcapsules in a variety of ways: as a component of the polyelectrolyte shell or in the internal cavity of microcapsules. The LbL method allows the integration of CeO_2_ nanoparticles by replacing one of the polyelectrolyte layers [101]: bare CeNPs in solution have a positive ζ-potential and thus can replace a polycation layer during microcapsule synthesis; conversely, carboxylic (citric, polyacrylic) acids normally used for the stabilization of cerium oxide nanoparticles provide a negative ζ-potential, and these particles can be incorporated into the microcapsule shell in place of one of the polyanion layers. The experiments in vitro on B50 neuroblastoma cells confirmed nanoceria delivery into the cell, so that polymeric LbL microcapsules can be considered to be an efficient intracellular delivery system for therapeutic nanoparticles [102].

Recently, CeNPs were incorporated into LbL polyelectrolyte microcapsules as a protective shell for an encapsulated enzyme (luciferase of *Photinus pyralis*), preventing its oxidation by hydrogen peroxide—the most abundant type of reactive oxygen species (ROS) [72]. Popov et al. demonstrated that the protective effect depends on CeO_2_ loading in the shell: at low concentrations, CeNPs only scavenge ROS, while a higher CeO_2_ content leads to a decrease in access for both ROS and the substrate to the enzyme in the core; by varying the nanoparticle concentration in the microcapsule, it is possible to control the level of enzyme shielding—from ROS filtering, to complete blocking. The optimal concentrations of ceria-containing microcapsules for human mesenchymal stem cells have been shown to be in the range of 1: 10 to 1: 20 cell-to-capsules ratios. Popov and co-workers revealed the molecular mechanisms of CeNPs-loaded LbL microcapsules’ radioprotective action on mesenchymal stem cells by assessing the level of intracellular ROS, as well as by a detailed 96-gene expression analysis, featuring genes responsible for oxidative stress, mitochondrial metabolism, apoptosis, inflammation etc. Ceria-containing microcapsules have been shown to provide an indirect genoprotective effect, reducing cytogenetic damage in irradiated cells [74]. Upon cellular uptake, CeNPs-loaded microcapsules degraded due to the action of intracellular enzymes and released content into cytoplasm. 

For the antioxidant protection of cells, Abuid et al. synthesized alginate microbeads containing β-cells, where two to 12 alternating layers of CeNPs/alginate were assembled onto alginate microbeads using a LbL technique [80]. These CeNPs/alginate coatings protected cells from ROS-mediated cellular dysfunction, with 12 layers of coatings providing complete preservation of cells’ metabolic activity, glucose stimulated response and antioxidant activity after ROS exposure. Ceria-containing LbL antioxidant coatings have shown great promise in both cellular implantation and in areas where mitigation of chronic inflammation is desired. Implantable drug delivery systems have been designed by Sedighi et al. to achieve controlled and sustained drug release [73]. Curcumin as a model drug was loaded into porous silicon containers (8.94 ± 0.72% w/w); then the containers were capped with cerium oxide nanoparticles for drug protection and release prolongation following layer-by-layer surface coating of the container with anionic (alginate) and cationic (chitosan) polymers, thus providing pH sensitivity. The presence of biopolymers increased both the biocompatibility and pH-dependent performance of the implantable container. The release profile confirmed that functionalization with CeNPs prevented burst-like blowout of a model drug.

**Figure 5:**
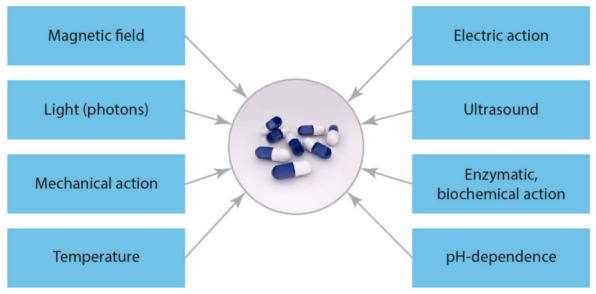
Smart release stimuli (single/dual/multi) for advanced drug delivery via microencapsulation.

The main smart release stimuli for advanced drug delivery via microencapsulation are shown in Figure 5.

## 6. Ceria Additives Improving Implants’ Biodegradability

The major factors affecting biomedical polymeric composites’ degradation rate are: polymer properties (chemical structure, molecular weight, crystallinity), surface properties (wettability, porosity), filler properties (sharp-dimension, functionalization, content), processing (morphology, interconnectivity, pore size), while polymeric composites’ degradation rate affects material functions, cellular response, drug release and biocompatibility [103]. Biodegradable biocompatible polymeric materials are materials that can degrade in a biological environment after a certain time, with the formation of non-toxic products that are excreted by the body or absorbed by it. The most promising areas of application of these materials in medicine include biodegradable suture threads, matrices for drug delivery to certain organs and cardiovascular, dental and orthopedic surgical temporary fasteners [104]. 

Mehta and colleagues developed microcarriers for encapsulating both nanoceria and metabolic enzymes, namely superoxide dismutase (SOD), using biocompatible and biodegradable poly(lactic-co-glycolic acid) (PLGA) [76]. Cellular uptake studies were conducted, along with a cytotoxicity assay. The antioxidative properties of PLGA-nanoceria-SOD particles were confirmed by adding H_2_O_2_ to cell culture and imaging with fluorescent markers of oxidative stress. The results obtained suggested that PLGA is a suitable encapsulating carrier for the simultaneous delivery of nanoceria and SOD together, and that this combination effectively reduces oxidative stress in vitro [76]. Aliphatic polyesters such as polylactide (PLA), poly(glycolides) (PGA) and poly(ɛ-caprolactone) (PCL) possess high biodegradability and biocompatibility [103]. The tunable degradation of poly(lactic-*co*-glycolic acid) (PLGA) makes it a candidate material for the encapsulation and delivery of active substances and drugs (for example, for SOD protecting by CeNPs [76]). Conversely, polyurethanes (PU) are slowly degradable or completely lack degradability. It has been shown that PU destruction in the body occurs via both non-enzymatic hydrolysis and an enzymatic, cellular pathway [105]. Phua et al. observed that proteolytic enzyme urease degraded segmented, cross-linked medical polyester Biomer^®^ at 37 °C in vitro after just a few months. Recently, Korschelt et al. demonstrated the urease-like activity of ceria nanorods [106], which suggests the possibility of controlling the rate of degradation of polyurethane items in the body by ceria nanoparticles.

## 7. Ceria-Containing Antibacterial and Antiviral Multifunctional Materials 

### 7.1. Antibacterial Cerium Compounds in Wound Healing

Cerium species are well known to have bactericidal properties and promise antibacterial applications [107,108,109]. The first example of the practical use of cerium compounds for the treatment of burn wounds at the end of the 19th century (Flammacerium^®^ cream) was based on the reported antibacterial effects of Ce(III) salt, while Ce(IV) sulfate was also used as an antiseptic powder [107]. Cerium (III) nitrate enhances anti-bacterial effects and imparts anti-inflammatory properties to silver dressings (Acticoat™, Mepilex™ and Silverlon^®^) in a rat scald burn model [110]. Thill et al. reported the antimicrobial activity of CeNPs against *Escherichia coli* [111]; Pelletier et al. confirmed CeO_2_ antibacterial action in both Gram-negative *Escherichia coli* and Gram-positive *Bacillus subtilis* [112], whereas Fang et al. showed the antibacterial activity of CeNPs against *Nitrosomonas europaea* [113]. Zholobak et al., in their recent review [107], analyzed the mechanisms of the antibacterial activities of cerium oxide nanoparticles and contoured the perspectives of their practical applications. 

### 7.2. Antibacterial CeNPs in Biomedical Polymers

Antibacterial protection in tissue engineering (e.g., prevention of the contamination of wound dressings, artificial scaffolds and protheses, the formation of bacterial and fungal biofilms on catheters and cannulas etc.) is an actual problem; the major contributors to morbidity and mortality from wounds have been known to be bacterial contamination during treatment. Shabrandi et al. demonstrated the healing effect of chitosan-supported nano-CeO_2_ on an experimental excisional wound infected with *Pseudomonas aeruginosa* in rats [114]. Topical application of such composition on the infected wound enhances tissue’s total antioxidant capacity, reduces the bacterial count, accelerates the proliferation and migration of fibroblasts and keratinocytes and increases the hydroxyproline level and neo-vascularization scale of the healing wound. Unnithan et al. fabricated CeNPs-doped electrospun antibacterial composite mats (polyurethane blended with two biopolymers, cellulose acetate and zein) using the electrospinning process [81]. They showed that the inhibitory effects of the composite on Gram-positive and Gram-negative bacteria depend strongly on the CeNPs’ concentration. Polyvinyl alcohol/chitosan hydrogel supplemented with 0.5% of CeNPs showed good antibacterial activity after just 12 h (against methicillin-resistant *Staphylococcus aureus*) and healthy human dermal fibroblast viabilities up to five days (more than 90%) compared to the control group [62]. Chitosan and cellulose acetate polymer composites filled with CeNPs as potential wound dressing materials have also demonstrated good antibacterial characteristics [64]. Fei et al. developed ceria-functionalized gelatin polycaprolactone nanofiber (PCLNPNF) matrices using electrospun methods; the antibacterial property of the PCLNPNF showed remarkable activity against both gram-positive (*S. aureus*), and gram-negative (*P. aeruginosa*), bacteria. Additional in vivo experiments confirmed the antibacterial action of the nanocomposites. Fei et al. suggested that the newly fabricated PCLNPNF was a promising antibiotic alternative for withstanding future bacterial infections [115]. Other bactericidal CeNPs-doped composites were fabricated by blending polyurethane with two biopolymers, cellulose acetate and zein; their antibacterial activity was confirmed with the most common pathogenic bacteria, such as *Escherichia coli*, *Klebsiella pneumoniae*, *Salmonella enterica* (Gram-negative), *Staphylococcus aureus* and *Enterococcus faecalis* (Gram-positive) [81]. The composite nanofibers demonstrated effective dose-dependent bactericidal activity against both the Gram-positive and Gram-negative bacterial strains. These CeNPs-doped composite nanofibers were considered to be a promising nanomaterial for highly efficient antibacterial treatment that could be used as a smart material for biomedical applications.

### 7.3. Antiviral CeNPs in Biomedical Polymers

Since the pioneering work of Zholobak et al. [116], demonstrating the antiviral ability of polymer-coated ceria nanoparticles, the antiviral/virucide properties of nanoceria have been studied intensively. Cerium oxide nanoparticles have been demonstrated to protect against the Herpes simplex virus 1 (HSV-1) in the RF cell line [117], and vesicular stomatitis Indiana virus (VSV) in L929 [116,117], EPT [116] and ST [118] cell lines. The activity of biogenic CeO_2_ nanoparticles against the Sabin-like poliovirus has been reported recently [119]. The COVID-19 pandemic has become a global health emergency and has stimulated much work in the development of antiviral materials. Under these conditions, nanoceria is a promising agent for fighting the SARS-CoV-2 coronavirus and management of the COVID-19 disease [120].

The antiviral activity of ceria nanoparticles can be associated with various phenomena, including direct virucidal action by ROS formation or oxidative stress induction via redox cycling under appropriate conditions (sunlight, UV-irradiation, the low pH of the microenvironment, etc.) [121]. The introduction of nanoceria into common polymers could decrease the survival rate of the virus (the ability of the virus to maintain infectivity) on the contaminated surface, including the surface of medical devices and gloves. Nanoceria in the polymer fibers can improve the antiviral protection of textile materials: masks, gowns and clothes. Nanoceria and film-forming water- or alcohol-soluble polymers make it possible to design a new class of virucidal disinfectant sprays or long-lasting sanitizers, forming a protective covering with prolonged action. 

Ceria-containing polymeric nano- and microparticles would be an effective adjuvant for antiviral vaccines [19,100,122] or drug carriers enhancing virus disease treatment (see above in Section 5.1).

## 8. Biosensing with Ceria-Containing Polymer Nanocomposites

Ceria-containing polymer nanocomposites are widely used for biosensing purposes. Such systems have been proposed for the detection of microorganisms and biologically important compounds. Thus, a facile label-free DNA sensor based on cerium oxide nanorods decorated with a polypyrrole nanoparticles (CeO_2_-NRs/Ppy-NPs) matrix has been developed for the detection of Salmonella [123]. The principle of operation of a biosensor proposed in this paper was based on the measurement of electrochemical impedance characteristics of single-stranded DNA (ssDNA) covalently immobilized onto a modified microelectrode, with the use of [Fe (CN)_6_]^3−/4−^ as a redox probe. The biosensor exhibited good linearity within the range of 1.0 × 10^−9^ mol L^−1^ to 1.0 × 10^−6^ mol L^−1^ with a sensitivity of 14.7 × 10^6^ Ω/mol L^−1^ cm^−1^; the detection and quantification limits were 2.86 × 10^−7^ mol L^−1^ and 9.56 × 10^−7^ mol L^−1^, respectively. 

Another example in this field is provided by Sun et al. [124], who studied an electrochemical biosensor based on a molecularly imprinted polymer (MIP) film for glycoprotein ovalbumin detection. In this biosensor, nanoceria was used as a redox active catalytic amplifier. Based on the results obtained, which demonstrated the linear response of the sensor within an analyte concentration range of 1 pg/mL to 1000 ng/mL, with a relatively low detection limit of 0.87 pg/mL, it was concluded that the proposed platform possessed good potential for clinical diagnostics and other related fields. Fu et al. engineered a novel electrochemical acetylcholinesterase biosensor, modified with cerium oxide-chitosan and ordered mesoporous carbon-chitosan, using a screen-printed carbon electrode fabricated to detect organophosphorus pesticides, utilizing good redox properties and a larger electron transfer rate of nanoceria [125]. Generally, nanoceria, along with Fe_3_O_4_ and gold nanoparticles, are often used for developing enzyme-mimicking nanomaterials (nanozymes), which are more cost-effective and robust than protein enzymes. There are multiple reports on biosensor devices in this field. For example, in order to improve the specificity of nanozymes, MIPs were grown on the surface of nanoceria, possessing oxidase-like activity, to create substrate binding pockets [126]. As a result, nearly a 100-fold increase in specificity was achieved. Selective substrate binding was further confirmed by isothermal titration calorimetry. It was concluded that the developed hybrid materials were highly promising for applications in biosensing and drug delivery. 

Another example is given by a conductive nanocomposite fabricated via the incorporation of CeO_2_ in a carbon matrix by the co-assembly of cerium nitrate, resol and a triblock copolymer, resulting in the formation of mesoporous ceria-carbon [127]. Glucose oxidase was subsequently immobilized in the vacant pores of the mesoporous ceria-carbon, using glutaraldehyde crosslinking to prevent enzyme leaching from the matrix. Hydrogen peroxide generated by the catalytic action of glucose oxidase was rapidly converted into hydroxyl radicals by the catalytic action of CeO_2_, which induced subsequent anodic oxidation of Ce^3+^ into Ce(OH)_2_^2+^ or Ce(OH)_4_ with the anodic current. The same electrochemical principle was realised in a biosensor fabricated by immobilizing horseradish peroxidase on polyaniline-cerium oxide nano-composite film onto indium-tin-oxide (ITO) coated glass substrate [128]. Chitosan nanocomposites reinforced with nanoceria-decorated reduced graphene oxide may also be used as an effective glucose oxidase immobilizer and bio-sensing matrix for glucose determination [129]. 

Paulter et al. emphasized that the nanozyme oxidase-like properties of nanoceria may be explored more efficiently through not only pH adjustment, but also the adsorption of anions such as phosphate and citrate [130]. It was pointed out that nanoceria bind DNA via the DNA phosphate backbone in a sequence-independent manner, inhibiting its oxidase activity. The ability of nanoceria to probe DNA was utilized by Wang et al. [131], who created an electrochemical DNA biosensor consisting of a composite film containing CeO_2_-ZrO_2_ hollow nanospheres and chitosan deposited on a gold electrode. It was shown that the target DNA could be quantified over a wide dynamic range of 1.63 × 10^−13^–1.63 × 10^−8^ M, with a detection limit of 1.0 × 10^−13^ M using methylene blue as an electrochemical indicator.

In the last decade, smart materials for colorimetric biosensing, including gold nanoparticles, magnetic nanoparticles, cerium oxide nanoparticles, carbon nanotubes, graphene oxide and conjugated polymers, have been developed [132]. The color of nanoceria is changed to yellow by the hydrogen peroxide generated during glucose oxidation by gold nanoparticles. Using this colorimetric sensor effect, it was proved that the adsorption of small molecules such as citrate does not deactivate gold nanoparticles, while the adsorption of polymers, including serum proteins and high molecular weight polyethylene glycol, inhibits glucose oxidation [133]. Further examples of nanoceria-containing biosensors have been given by core-shell structured poly(3,4-ethylenedioxythiophene) (PEDOT) composite nanofibers as peroxidase-like catalysts for the colorimetric detection of hydrogen peroxide [134] and fluorescent ATP-Ce(III)-tris(hydroxymethyl)aminomethane coordination polymer nanoparticles being oxidized to non-fluorescent ATP-Ce(III)-tris(hydroxymethyl) aminomethane by hydrogen peroxide [135]. 

## 9. Other Biomedical Applications and Future Trends of CeNPs-Polymer Composites 

**Figure 6:**
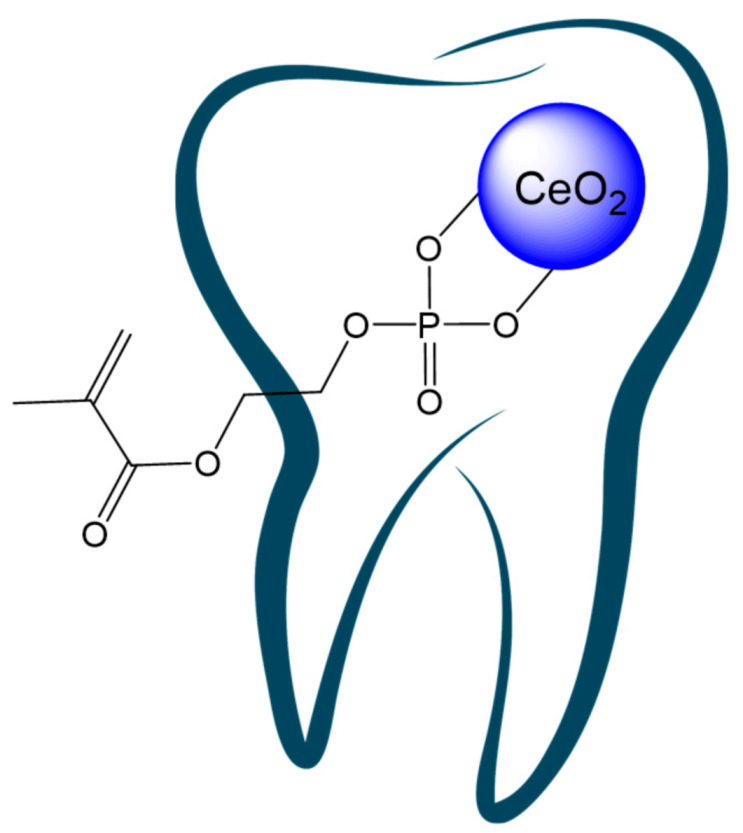
Cerium oxide nanoparticles modified with methacrylate phosphate for possible use as a filler for curing dental polymer compositions.

Ceria-containing polymer composites could be useful for various implants and medical devices (including hearing aids and orthodontic and orthopedic products, etc.). For instance, CeNPs have been shown to protect human dental stem cells from oxidative insult [136] and have been used as a filler to tune the radiopacity of dental polymeric adhesive (methacrylate resin) to meet the requirement of ISO 4049 [137]: the results obtained have shown that CeO_2_ at 4.32 vol.% exceeds the radiopacity threshold value. CeNPs have also demonstrated antibacterial activity against dental bacteria (including *Streptococcus mutans* [138]) responsible for cariogenic processes. Thus, CeNPs show promise in orthodontal practice (including dental prosthetics, or as a filler for organic-inorganic cements and curing dental polymer compositions, Figure 6).

Medical device-induced thromboses (including stent thrombosis [139]) have become a major concern in clinical practice. A blood clot is a common cause of failure of blood-contacting medical devices, such as vascular grafts, heart valves, venous catheters and ports [140]. The ability of cerium ions to prevent blood coagulation and to increase the clotting time of normal human plasma is an established fact [141]. In turn, the use of CeNPs as an anticoagulant is restricted due to the toxicity of soluble cerium species. The incorporation of antithrombogenic CeNPs into polymeric material of venous catheters and cardiovascular prostheses, or the coating of coronary stents with CeNPs, could help in the design of blood-compatible medical items.

**Figure 7:**
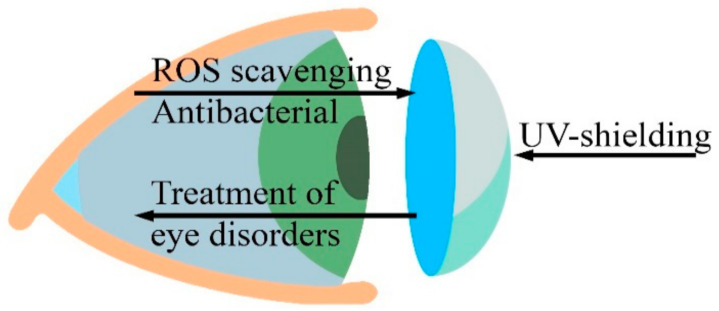
CeNPs can provide additional advantages for contact lens polymeric materials.

Based on their biomedical properties and safety, CeNPs can be used in the treatment of several eye disorders [142]. Chen et al. showed that nanoceria decrease the intracellular concentration of reactive oxygen species in primary cell cultures of rat retinas, as well as prevent loss of vision caused by light-induced degeneration of photoreceptor cells [143]. In vitro cytotoxicity studies have shown that CeNPs could be safe for lens cells and could provide a new therapy for cataract treatment [142]. The retina is highly susceptible to oxidative stress because of its high oxygen consumption and high metabolic activity associated with exposure to light. Kyosseva and McGinnis emphasized that cerium oxide nanoparticles can act as an antioxidant in rodent models of age-related macular degeneration and inherited retinal degeneration and thus may provide a novel therapeutic strategy for the treatment of human eye diseases [144]. Contact lenses are used for the correction of refractive errors. CeNPs, due to their high transparency in the visible light range of spectra, as well as to their antioxidant, therapeutic, antibacterial and UV-shielding properties, could be a good candidate as a filling material for contact lenses (Figure 7) or ocular implants (e.g., intraocular lens/corneal substitutes, etc.).

Polymeric surgical glues are widely used to seal small holes (sealants), to stop bleeding from damaged tissues (hemostatic agents) or to bond separated tissues (surgical adhesives) in cardiovascular, thoracic, urologic, gastroenterological, gynecologic, plastic and orthopedic surgeries, and neurosurgery [145]. Cerium oxide nanoparticles in polymeric compositions could improve the chemical properties of glues (e.g., curation time), as well as increase the physical (mechanical strength, water resistance) and biomedical (tissue adhesion, lesion healing) properties of cured glue.

The development of artificial muscles has been a modern trend in recent years, and currently such materials can induce strains exceeding 100%. Dielectric elastomer electroactive polymers are among the materials that are promising for such applications [146]. The dielectric properties and conductivity of polymeric materials can be tuned by nanocrystalline cerium oxide, as demonstrated for PVA/PVP hydrogel [147], as well as for polyaniline [148], polystyrene [149] and polyethylene [150] matrices.

Gene therapy has become an actual protocol for treating certain human diseases. Various types of cationic polymer and DNA-trapping degradable polymer nanoparticles have been assessed for their potential applicability to this field [151]. CeNPs are a promising carrier and analogue of restriction enzymes in gene therapy [19], and thus they can be used as an active component in such polymeric vehicles.

Polymers play an important role in the development of drug carriers for cancer treatment. Polymeric nanocarriers with conjugated or encapsulated anti-cancer drugs, also known as polymeric nanomedicines, form many different architectures, including polymer-drug conjugates, micelles, nanospheres, nanogels, vesicles and dendrimers [152]. Nanocrystalline ceria is a well-known medication in cancer theranostics [121]. CeNPs have been successfully used to improve cancer photodynamic therapy (PDT), which often lacks selectivity. Reactive oxygen species (ROS), being generated regardless of the disease microenvironment, may induce significant damage to normal tissues. Hybrid nanoparticles of an NIR-absorbing semiconducting polymer, acting as the NIR fluorescent PDT agent, and CeNPs, able to decrease and increase ROS generation in physiologically neutral and pathologically acidic environments, have been used in PDT experiments for cancer therapy in a murine model [153]. It was found that the modification of the PDT agent with CeNPs did not affect its NIR fluorescence imaging ability, since nanoceria are optically inactive. Nevertheless, the nonspecific damage to normal tissue under NIR laser irradiation was drastically reduced and PDT efficiency significantly improved when a CeO_2_-containing polymeric composite was used instead of a bare polymer. 

The skull is one of the most important bone structures in vertebrates, which provides protection and mechanical support for the brain and accessory organs. Many skull defects can fatally threaten human life, but some of them can be repaired by cranioplasty, the surgical filling of the defective area with synthetic materials, such as polymers. Polymethyl methacrylate (PMMA) and polyetheretherketone (PEEK) are among the most popular polymers for use as neurosurgery implants and as a promising alternative to traditional metallic implants. Pristine or filled PMMA-based bone cement is the most widely adopted in vertebroplasty; bioactive glasses and hydroxyapatite represent the most common type of fillers for functionalizing and reinforcing the cement; metal nanoparticles (silver, gold) and copper doped tricalcium phosphate have shown an antibacterial effect [154]. However, PMMA bone cement can be chemotoxic to the dura mater and cerebral cortex by releasing the potentially neurotoxic methyl methacrylate monomer [155]. Ceria nanoparticles possess not only antibacterial, but also neuroprotective, properties; they improve the histopathology and morphological abnormalities of dorsal root ganglion neurons [156] and they have been shown to be useful as a medicine for neurodegenerative diseases [157] or therapeutic intervention after a stroke [158]. Obviously, CeNPs would be a suitable filler for cranioplasty polymer materials, decreasing polymer toxicity and enhancing brain injury healing rate.

A less discussed problem in the use of CeNPs-filled polymeric composites as implants or smart materials is the effect of ceria nanoparticles on polymers’ permeability, primarily their permeability to physiological fluids. Transport phenomena and mass transfer in polymers are very complex problems to understand and require consideration of both porous media and filler properties, along with the nature of permeant and interface effects. Some advances in the permeability of porous fibrous media have been discussed elsewhere [159,160,161] but there is a strong need for further progress in regard to composite materials containing CeNPs.

Cerium oxide nanoparticles can enhance the radiocontrast, radiopacity and radioprotection properties of polymeric material. They can be used for the visualization of implants, shielding/protection of healthy tissue against radiation (e.g., during cancer radiotherapy) or tuning of radiophysical/radiochemical processes in living cells. For example, internal radiation therapy uses temporary or permanent brachytherapy implants (catheters or applicators). CeNPs-releasing bulk polymer materials or polymer coatings of such implants can help to protect healthy tissue during treatment. Ouyang et al. demonstrated that cerium oxide nanoparticles can be employed for radioprotection during accelerated partial breast irradiation (*MammoSite* Breast Brachytherapy), using a new design with balloon applicators coated with CeNPs for sustained/controlled in situ release from within the lumpectomy cavity [162]. In turn, the ^68^Ge/^68^Ga generator has great potential for clinical positron emission tomography (PET) imaging; however, because of the unavailability of a suitable sorbent material, commercially available generators are not yet suitable for the preparation of ^68^Ga-labelled radiopharmaceuticals. Chakravarty et al. discovered new CeNPs-polyacrylonitrile composite-based advanced sorbent material, which has been shown to possess the best characteristics for the development of a clinical grade generator of these radiopharmaceuticals [163].

Thus, analysis of current trends makes it possible to open new perspectives for numerous applications of biomedical items made from polymers filled with cerium oxide nanoparticles.

## 10. Conclusions

In this review, the authors have attempted to summarize the current state of the art and prospects for the use of cerium oxide nanoparticles in advanced polymer composites for biomedical applications. Currently, polymeric materials have become widespread in various biomedical fields. Despite their rapidly expanding use, there remain some acute, unresolved issues, such as: the rejection of polymeric transplants due to foreign body reaction; the insufficient healing rate of wound dressings due to low cell proliferation and tissue inflammation; bacterial contamination of implantable items and biofilm formation, etc. 

Due to their unique properties, nanoceria show great promise for use in regenerative medicine and tissue engineering; nanoceria are a well-known oxidoreductase- and phosphatase-like nanozyme, and the first inorganic mitogen ever reported; they stimulate the proliferation of cells and accelerate the healing of lesions in vivo. Nanoceria reduce inflammation and autoimmune response; they possess antimicrobial and anti-biofilm properties and have many other benefits that are in high demand for biomedical applications.

Studies included in this review have shown that nanoceria-containing polymeric composites combine the advantages exhibited by each component of the material and reduce the intrinsic drawbacks of both nanoceria and polymeric matrices (see also Table 2). One of the main focal points of the review was to demonstrate the positive effect of nanoceria on advanced biomedical polymeric materials, including the enhancement of UV-resistance and UV-protective properties, the adhesion of biomolecules and cells, biocompatibility, etc. Table 2 shows that nanoceria-containing polymeric composites make it possible to combine the advantages exhibited by each component of the material and enhance the biomedical potential of both nanoceria and polymeric matrices.

The development of ceria-polymeric composites is expected to mitigate possible risks and speed up nanoceria introduction in the field of biomedicine. It is because of these properties that nanoceria-polymer composition will very likely be among the first approved nanoceria-containing products in clinical practice.

## Figures and Tables

**Table 1:** Composition and biochemical applications of ceria-polymeric composites.

Polymer	CeNPs Characteristics and Content (*w/v*%)	Nanocomposite Products	Applications	Results	Ref.
**Tissue engineering**					
Cellulose	Cubic CeNPs, 3.2–32 nm; 300 mL of water containing 1- or 5-mM cerium nitrate and 1% cellulose was used as a precursor.	Three-dimensional scaffolds	Tissue engineering	Nanobiocomposites are not cytotoxic to HeLa cells at a concentration as high as >1 mg·mL^−1^ and scavenge ROS.	[48]
PCL	Size 9–16 nm; 0.5%, 1%, 2% and 3%.	Electrospun fiber scaffolds	Tissue engineering	In vitro (MSC): higher cell adhesion and proliferation were evident relative to bare PCL. In vivo (rats): high cell proliferation rate and blood formation. Angiogenesis was activated by HIF-1α, as shown by the upregulation of VEGF expression in the nanocomposite scaffolds.	[55]
PL	CeNPs having different Ce^4+^ and Ce^3+^ ratios	Scaffold/artificial-niche	Tissue engineering	Mesenchymal stem (MSCs) and osteoblast-like (MG63) cells were cultured on PL/CNP surfaces with Ce^4+^- or Ce^3+^-enriched regions. Despite cell type (MSCs and MG63 cells), different cerium valence state regions promoted or inhibited cell spreading, migration or adhesion behavior, resulting in rapid or slow cell proliferation.	[56]
PL	~5 nm, oleylamine-capped CeNPs, up to 10 wt%: cancellous bone impregnated by PL + CeNPs	Scaffolds	Bone tissue engineering	In vitro: coculture of endothelial progenitor cells and MSC on scaffold supplemented with CeNPs showed the improvement of cell viability and the differentiation process for endothelial progenitor cells. In vivo (mice): higher level of vascularization for scaffold supplemented with CeNPs in comparison with the bare scaffold.	[57]
Gelatin	CeNPs by AlfaAesar as a 20% aqueous solution at acidic pH, with nominal size <5 nm. 15% gelatin and 1 mg/mL CeNPs were used as a precursor, GPTMS as a cross-linker	Electrospun fiber scaffolds	Neuronal tissue engineering and regenerative medicine	The scaffolds demonstrate strong antioxidant properties and beneficial multi-cue effects in terms of neurite development and alignment on neuron-like SH-SY5Y cells.	[58]
Gelatin/alginate	Mean diameter 35.5 nm, zeta potential −12.35 ± 1.39 mV; 100 μg/mL, 500 μg/mL and 1000 μg/mL	Scaffolds	Bone regeneration	Highest mesenchymal stem cells (MSCs) proliferation rate was observed for 1000 μg/mL CeNPs scaffolds; application of the scaffolds resulted in enhanced osteogenic differentiation of MSCs, as well as free radical scavenging.	[59]
POC	<25 nm particle size (Sigma-Aldrich), 10 or 20 wt% relative to POC	Scaffolds	Bone tissue regeneration	Scaffolds are biocompatible and supported cell attachment, proliferation, mineralization and infiltration. They possess protective properties against ROS via the reduction in cytotoxicity, improving mineralization of osteoblast cells in vitro. Cells are able to infiltrate through the scaffolds, the surrounding tissues elicit a minimal immune response. Nanocomposite scaffold system is capable of supporting bone-remodeling processes while providing a protective free radical scavenging effect.	[60]
PLGA	CeNPs size ~5 nm; 20 mg of CeNPs in 200 mg of PLGA	Microparticles and scaffolds	CeNPs delivery, tissue engineering including bone remodeling and regeneration	The release kinetics of CNPs from PLGA matrix was investigated under acidic, basic and near-neutral pHs. Superoxide dismutase (SOD) mimetic activity was retained in released CNPs for a long period of time (∼90 days). PLGA encapsulated CeNPs showed excellent biocompatibility.	[61]
**Wound healing/dressing**					
Chitosan/PVA	zeta potential 50 mV, ∼5 nm in diameter, 0.5% and 1%	Hydrogels	Wound healing	Enhanced cell compatibility and survival, antibacterial activity against MRSA	[62]
PHBV	8.6 ± 3.8 nm in diameter (TEM); 0.5%, 1%, 2% and 4%	Electrospun membranes	Diabetic wound healing	In vitro: For less than 1% w/w of CeNPs content, human mammary epithelial cells adhered parallel to individual fibers; for higher CeNPs content, cells started to flatten and spread over the fibers. In ovo: enhanced blood vessel formation. In vivo (rats): promotes healing of diabetic wounds	[63]
Chitosan/cellulose acetate	<25 nm particle size (Sigma-Aldrich), 0.1% and 1%	Films	Wound dressing	Good water vapour transmission rates (WVTR) and water vapour permeability (WVP) values, antibacterial behavior for *S. aureus* and *E. coli*.	[64]
PCL/gelatin (1:1)	<25 nm particle size, 1.5, 3 and 6%	Electrospun films	Wound dressing	In vitro: 1.5% CeNPs exhibited the highest cell proliferation with L929 cells. In vivo: 1.5% CeNPs accelerated wound healing compared with the sterile gauze.	[65]
PCL/gelatin (1:1)	~42 nm in size, zeta potential 30.8 mV. The nanofibers were fabricated from a polymer solution of 10% *w/v* PCL, 20% *w/v* gelatin and 25% *v/v* 30 mM CeNPs	Electrospun fibers	Wound healing	Enhanced proliferation of 3T3-L1 cells (by ~48%), ROS scavenging ability, three-fold increase in the viability and proliferation of cells.	[66]
Gelatin	2.5–6.5 nm in size. From 50 μg/mL to 500 μg/mL dispersed into gelatin solution (5%, w/v), optimal concentration 250 μg/mL	Composite hydrogels	Wound healing	In vitro: 250 μg/mL provided 86 ± 1.4% cell viability and increased bound water content (swelling ratio was three-fold to that of native gelatin). In vivo (rats): more infiltration of leukocytes and larger deposition of collagen, the wound was healed in 12 days.	[67]
GelMA-DOPA	10–30 nm in size (US Research Nanomaterials), 100.0 μg/mL	Sprayable hydrogel	Wound dressing	Hydrogel provided a multifunctional wound dressing with desired antimicrobial, ROS-scavenging, adhesive, and degradative properties both in vitro and in vivo.	[68]
PCL	Mesoporous CeO_2_ nanorods, 5–25%, optimal 15%	Nanomembranes	Cutaneous wound healing	High antimicrobial activity against *E. coli* and *S. aureus*, strong wound healing effect, good biocompatibility.	[69]
Zwitterionic cryogel of CBMA or SBMA and HEMA	CeNPs size range of 3–5 nm; 68 μL of aqueous 36.6 μM FITC-labelled CeNPs were added to 250 μL of gel prior to polymerization	Injectable gels	Wound healing	The gels speed up diabetic wound healing and significantly reduce inflammation.	[70]
Gelatin/oxidized dextran	Particle size < 50 nm, 430 ug in 1 mL of gel	Hydrogel dressings	Wound healing	Prolonged drug (curcumin) release (∼63% in 108 h), accelerated cell migration, significant antioxidant and anti-inflammatory activity in vivo (∼39%).	[71]
PAA/curcumin	220 by 30–75 nm CeNPs; 0.1 mM, 0.2 mM and 0.4 mM	Hydrogel dressings	Scarless healing of injury	In a full-thickness acute wound healing model of rat, a single application of dressing demonstrated higher wound healing efficacy (78%) and negligible scarring in 7 days. Enhanced cell proliferation, higher collagen content, advanced wound maturity, re-epithelialization and granulation tissue formation were observed.	[50]
**Drug delivery**					
PArg/DS	Citrate-stabilized CeNPs, 4–7 nm, ζ-potential ~–40 mV	LbL microcapsules	Drug delivery	CeNPs provide “active” protection of loaded content (luciferase enzyme) against hydrogen peroxide and “passive” shielding against small molecules.	[72]
Alginate/Chitosan	Citrate-stabilized CeNPs, diameter ~5 nm, ζ-potential −16.99 ± 2.72 mV	LbL-coated silicone containers	Drug delivery	CeNPs functionality prevents burst blowout of model drug (curcumin).	[73]
PArg/DS	Citrate-stabilized CeNPs, 2–2.5 nm, negative ζ-potential	LbL microcapsules	Drug delivery, radioprotection	CeNPs microcapsules provide enhanced cellular internalization and good radioprotection.	[74]
PEG/PLGA	Mostly uniform spherical CeNPs 5–10 nm in size	~40 nm nanoparticles	Cerebral ischemic therapy, brain targeted drug delivery	10 mg/kg concentration resulted in 60–78% lessening of focal ischemia in middle cerebral artery occlusion model of brain stroke.	[75]
PLGA	Diameter of 2–3 nm; 1 μM of CeNPs was suspended in 2.5% aqueous PVA solution containing 40 mg of PLGA as a precursor	Microparticles	CeNPs and drugs co-delivery	PLGA is a suitable encapsulating carrier for simultaneous delivery of nanoceria and SOD. This combination effectively reduces oxidative stress in vitro.	[76]
**Other biomedical applications**					
PVA	0.5, 1.5 and 3%	Electrospun mats of nanocomposite hydrogels	Various biomedical applications	Better platelet adhesion and accelerated wound healing	[77]
TPU	CeNPs size ∼60 nm; 0.1–0.7 wt%		Various biomedical applications	Enhanced blood compatibility, cell viability, chemical resistance, mechanical and thermal properties of TPU.	[53]
Alginate	Dextran-coated CeNPs, 2.7–9 nm radius (23.8% polydispersity); 0.1, 1.0 and 10 mM CeNPs in hydrogel.	Composite hydrogel microcapsules	Cellular transplantation	Cytoprotection of encapsulated insulin-producing MIN6 beta cells from free radical attack. No cytotoxicity up to 10 mM CeNPs.	[49]
PLGA	5 to 8 nm in size; 5, 10 and 20 wt%	Hybrid 2D polymeric-ceramic biosupports	Regenerative medicine	Better murine derived cardiac and mesenchymal stem cells’ proliferative activity is observed for CeO_2_ polymer composites with respect to either TiO_2_-filled or unfilled PLGA films.	[78]
PL/Gelatin	Polyhedral nanoparticles 5–10 nm in size; 0.25%,0.5% and 1%	Electrospun fibro-porous membranes	Scaffolds for angiogenesis	Good hydrophilicity, water absorption and improved mechanical properties; scaffolds were shown to be biocompatible both in vitro (somatic hybrid endothelial cells) and in vivo (chick embryo angiogenesis assay); pro-angiogenic activities of the scaffolds are comparable to VEGF.	[79]
Alginate	Particle size < 5 nm, 20 wt% in H_2_O, pH~4 (Sigma Aldrich)	LbL-coated alginate microbeads	Biomedical implants, including cellular transplantation	12 layers of CeNPs/alginate provided complete protection to the entrapped beta cells from exposure to 100 μM H_2_O_2_, with no significant changes in metabolic activity, oxidant capacity or insulin secretion dynamics, when compared to untreated control.	[80]
PU with CA/Zein	CeO_2_ nanofibers were composed of nanoparticles ca.10–20 nm in size; 10%	Electrospun fiber mats	Antibacterial smart material	Composite nanofibers demonstrated notable toxicity against *Escherichia coli*, *Klebsiella pneumoniae*, *Salmonella enterica* (Gram-negative), *Staphylococcus aureus* and *Enterococcus faecalis* (Gram-positive) strains.	[81]

CA = Cellulose acetate; CBMA = 3-[[2-(Methacryloyloxy)ethyl] dimethylammonium] propionate; CeNPs = Ceria nanoparticles; GPTMS = (3-glycidyloxypropyl) trimethoxysilane; DOPA = dopamine; HEMA = 2-Hydroxyethyl methacrylate; GelMA = gelatin methacryloyl; PAA = Poly(acrylamide); PArg = poly-L-arginine; DS = dextran sulfate; PCL = Poly(ε-caprolactone); PEG = Polyethylene glycol; PHBV = Poly(3-hydroxybutyrate-co-3-hydroxyvalerate); PL = Poly-(l-lactide); PLGA = Poly(lactide-co-glycolide); POC = Poly(1,8-octanediol-co-citrate); PU = Polyurethane; PVA = Polyvinyl alcohol; SBMA = [2-(methacryloloxy) ethyl] dimethyl-(3-sulfopropyl) ammonium hydroxide; TMC = Trimethyl chitosan; TPU = Thermoplastic polyurethane.

**Table 2:** Improvements to the properties of ceria nanoparticles and polymers by the development of ceria-polymeric composites.

**Improvement of Ceria Nanoparticles’ Properties**	
Physical, chemical	Decreased CeNPs solubility and free Ce-ions leakage; Local concentration control; Surface charge control; Stimuli-related release control; Tunable microenvironment for anti/prooxidant activity control.
Biomedical	Decreased toxicity: Decreasing Ce-ions’ toxicity; Reducing phagocytosis; Preventing cell membrane damage. Easy-to-remove materials; Impaired clearance.
**Improvement of polymer properties**	
Physical	Tunable mechanical, thermal and electric properties; UV-resistance, UV-protection and shielding; Radiodensity/radiopacity; Roughness and surface energy control; Wettability, swelling and solubility control; Porosity and permeability control (liquids, gases, water vapors).
Chemical, biochemical	Enhanced chemical resistance, decreased erosion/corrosion, prolonged durability; Antioxidant properties: Free radicals scavenging; ROS decomposition; Oxygen buffering. Redox balance control; Enhanced biomolecules adhesion; Tunable scaffold mineralization; Mitigation of proinflammatory cytokines level.
Biomedical	Enhanced cell adhesion, proliferation, migration and tissue repair; Faster healing rate; Better biocompatibility; Decreased inflammation; Decreased foreign body reactions and rejections; Bactericide/bacteriostatic, fungicide, virucide activity; Enhanced implants visualization (radiocontrast); Biodegradability control.

## Data Availability

Not applicable.
